# Study on the Enhancement of Recovery Rates in Heterogeneous Dense Reservoirs Using Foam Drives Stabilized by Low-Surface-Tension Nanoparticles

**DOI:** 10.3390/molecules31142488

**Published:** 2026-07-16

**Authors:** Zhe Wang, Shanfa Tang, Yu Xia, Zequn Chen

**Affiliations:** 1 College of Petroleum Engineering, Yangtze University, Wuhan 430100, China; wz491324707@163.com (Z.W.);; 2 Hubei Provincial Key Laboratory of Oil and Gas Drilling and Production Engineering, Wuhan 430100, China; 3 State Key Laboratory of Low Carbon Catalysis and Carbon Dioxide Utilization, Yangtze University, Wuhan 430100, China

**Keywords:** low-permeability oil reservoir, foam flooding, low interfacial tension, enhanced oil recovery

## Abstract

Aiming at the problems of low permeability, strong heterogeneity, high salinity, poor injectivity of the target reservoir, and the inability of traditional water flooding to effectively improve the oil recovery rate, a low interfacial tension and salt-resistant nano-scale particle-stabilized foam flooding system (LITF-NSF) was developed to improve the recovery rate of low-permeability oil reservoirs. Through core dynamic plugging and injectivity experiments, single/dual-tube core displacement experiments, and static imbibition oil displacement experiments, the injectivity, plugging effect, imbibition effect, oil displacement mechanism, and optimal injection parameters of the LITF-NSF foam flooding system were studied from three aspects: injection pressure, slug volume, and gas–liquid ratio. The LITF-NSF foam flooding system has good injectivity in the target reservoir, with a resistance coefficient ranging from 1.086 to 15.468; the optimal injection parameters are a constant pressure difference of 5 MPa, a gas–liquid slug volume of 0.3 PV, and a gas–liquid ratio of 2:1. Under these optimal injection parameters, the subsequent water flooding recovery rate can be increased by 29.77%; it can produce a good plugging effect on the high-permeability reservoirs of the target reservoir with a permeability difference of less than 50, and can increase the comprehensive recovery rate of heterogeneous oil reservoirs by more than 10%; the static imbibition recovery rate is 4.38% higher than that of pure water without surfactant, and has a certain permeability-enhancing oil displacement effect. The LITF-NSF foam flooding system has good adaptability to the target reservoir environment and stable foam performance, which can effectively improve the subsequent water flooding recovery rate and has a good application prospect in chemical flooding for improving the recovery rate of low-permeability oil reservoirs.

## 1. Introduction

Currently, China’s terrestrial heterogeneous tight oil reservoirs are generally characterized by shallow reservoir thickness, low reservoir permeability, poor physical properties, high heterogeneity, and high mineralization. As the reservoir enters the medium-water-cut production phase, contradictions between plan and cross-sectional data have intensified, giving rise to issues such as low single-well production, rapid production decline and difficulties in maintaining stable production across the area. Consequently, there is an urgent need to identify new development methods to enhance crude oil recovery.

In conventional polymer-based chemical flooding, the high molecular weight of the polymers makes it difficult for them to penetrate the small pores and throat channels of low-permeability reservoirs, resulting in poor oil displacement efficiency [1,2,3,4]. In contrast to traditional water and gas flooding, foam flooding offers selective blocking that traps water without blocking oil, thereby improving crude oil recovery to some extent. However, it also faces challenges such as the inability to effectively strip residual oil adhering to rock surfaces [5,6,7,8,9,10]. In recent years, many researchers have investigated oxygen-depleted air foam flooding technology. Research findings indicate that oxygen-depleted air foam flooding combines the advantages of both foam flooding and air injection flooding; it allows for flow control and can also overcome issues such as gas migration and viscous flow, offering broad application prospects in reservoirs with varying permeability. Furthermore, when low-surface-tension surfactants are used as foaming agents, the resulting foam can dislodge residual oil adsorbed on the surfaces of small pores and rock walls in low-permeability reservoirs, yielding superior oil displacement performance [11,12,13,14,15,16,17,18].

Based on this, this study focuses on a heterogeneous tight oil reservoir in a certain block in China and proposes a low-interfacial-tension, salt-tolerant nano-particle-stabilized foam oil recovery system (Low Interfacial Tension Nano-Stabilized Foam, LIFT-NSF), This LIFT-NSF system can, to a certain extent, improve the overall recovery rate from heterogeneous tight oil reservoirs. Meanwhile, a study on the reservoir adaptability of the LIFT-NSF system was also conducted, aiming to provide theoretical guidance for the implementation of the LIFT-NSF system at actual oil fields by systematically evaluating the LIFT-NSF system’s plugging properties, injectability, and oil displacement and permeability enhancement effects in heterogeneous rock cores.

## 2. Experimental Section

### 2.1. Materials and Apparatus

The oil used in the experiment was crude oil from the target block formation, with a viscosity of 6.58 mPa·s and a density of 0.811 g/cm^3^ at a formation temperature of 55 °C. The water used in the experiment was formation water simulating that of the block, with a total salinity of 118,212 mg/L and a CaCl_2_ type; key ion composition: ρ(Ca^2+^) is 20,439 mg/L, ρ(Na^+^ + K^+^) is 2000 mg/L, ρ(Mg^2+^) is 415 mg/L, ρ(Cl^−^) is 78,813 mg/L, ρ(SO_4_^2−^) is 1172 mg/L, and ρ(HCO_3_^−^) is 73 mg/L. The experimental gas used was high-purity N_2_ produced by Wuhan Newrade Special Gases Co., Ltd., with a purity of up to 99.99% (by volume), which is located in Wuhan, China. The experimental low-surface-tension, salt-tolerant nano-particle-stabilized foam system LIFT-NSF (composed of 0.35% LS-12 (dodecyl hydroxysulphate) +0.08% LT-14 (tetradecyl amine salt) +0.03% carboxyl-modified hydrophilic nano-SiO_2_ (WB), with a foam index of 15,000 mL·min, and an interfacial tension between the liquid–gas of 6.46 × 10^−2^ mN/m), was independently developed by the School of Petroleum Engineering at Yangtze University, which is located in Wuhan, China. The experimental cores consisted of 17 small piston cores extracted from the target formation; the results of the physical property tests on the cores are listed in Table 1, and the specific composition of the LIFT-NSF foam system is listed in Table 2.

ISCO dual-chamber constant-pressure, constant-flow pump, HKY-N1 gas flow controller, core holder, Jiangsu Hai’an Petroleum Research Instrument Co., Ltd. Which is located in Nantong, China; TC-300 air pump, Hai’an Tuochuang Research Instrument Co., Ltd. Which is located in Nantong, China.

### 2.2. Experimental Methods

#### 2.2.1. Preparation and Evaluation of the LIFT-NSF Foam System

(1) Preparation of the foam system, specifically including the preparation of LS-12 foaming agent, LT-14 foam stabilizer, and modified nano-silica; the main preparation steps for each reagent are as follows:

(a) Preparation of LS-12 foaming agent, LT-14 foam stabilizer. Taking the synthesis of a di-dodecyl hydroxysulphate-type zwitterionic surfactant from dodecylamine, ethylenediamine, chloroethylsulphate, and epichlorohydrin as an example.

➀ Synthesis of sodium ethylenediamine diethylsulphate: Weigh out a specific mass of ethylenediamine and sodium chloroethylsulphonate in a ratio of n(ethylenediamine):n (sodium chloroethylsulphonate) = 1:2, place them in a flask, and add an appropriate amount of distilled water. React at a constant temperature of 85 °C for 14 h, maintaining the pH of the reaction system within the range of 9 throughout the process. Upon completion of the reaction, the solvent is removed using a rotary evaporator, followed by washing with anhydrous ethanol, vacuum filtration, and drying to obtain the product. The product yield is approximately 43 per cent.

➁ Synthesis of sodium B, N, N-di(3-chloro-2-hydroxypropyl) ethylenediamine diethylsulphate: Add a specific mass of the intermediate sodium ethylenediamine diethylsulphate (A) and an appropriate amount of distilled water to a flask and dissolve thoroughly; adjust the pH of the reaction mixture to 7 using formic acid. Add the corresponding mass of epichlorohydrin dropwise at a molar ratio of n(A):n(epichlorohydrin) = 1:2.3. Allow the reaction to proceed at a constant temperature of 30 °C for 3 h. Upon completion of the reaction, remove the solvent using a rotary evaporator to obtain the product. The product yield is approximately 48%.

➂ Synthesis of sodium C, N, N-di(3-chloro-2-hydroxypropane-N-dodecylamine) ethylenediamine diethylsulphonic acid: Add a specific mass of B (N, N-di(3-chloro-2-hydroxypropane) sodium ethylenediamine diethylsulphate) and an appropriate amount of distilled water to a flask, dissolve thoroughly and heat to 85 °C. Add the corresponding mass of dodecylamine according to the ratio n(B):n (dodecylamine) = 1:2, and react at a constant temperature for 12 h, maintaining the pH of the reaction system at 9. After the reaction is complete, the target product can be obtained following purification. The product yield is approximately 70 per cent. By simply varying the carbon number of the organic amine, corresponding dialkyl hydroxysulphates with dual hydrophobic carbon chains of different lengths can be prepared under similar reaction conditions.

(b) Preparation modified nano-silica. Silane coupling agents were used to graft-modify nano-silica particles. The experimental procedure for hydrophobic (homogeneous) modification of nano-SiO_2_ particles is as follows:

➀ Prepare an ethanol–water solution with a ratio of v (anhydrous ethanol):v (purified water) = 7:3. Then, add a specific quantity of nano-SiO_2_ to the prepared ethanol–water solution and sonicate to form a stable dispersion. Adjust the experimental temperature to 40 °C, place the thoroughly mixed dispersion in a magnetic stirrer, and stir for 12 h.

➁ Once stirring has been completed in accordance with the above steps, use a disposable pipette to slowly and uniformly add 1–2 mL of γ-(2,3-epoxypropyl) trimethylsilane with a purity of >99%. Maintain a constant temperature of 40 °C throughout the stirring process. Continue mechanical stirring for a further 24 h. Upon completion of stirring, remove the three-neck flask and centrifuge it directly using a centrifuge (5000 rpm, 15 min) to remove the supernatant, followed by four alternating centrifugation washes (with ethanol and purified water).

➂ The centrifuged product obtained in step ➁ was dried in a vacuum oven at 70 °C for 12 h, yielding homogeneous hydrophobic-modified nano-silica particles (with propyl hydrophobic groups).

(2) Evaluation of the foam system, specific evaluations include foam performance testing, oil–water interfacial tension testing.

(a) Foam performance testing:

The experimental procedure for evaluating the foaming properties of surfactants using the Ross–Miles method is as follows:

➀ Connect the outlet and return pipes of the constant-temperature water bath to the inlet (bottom) and outlet (top) pipes of the jacketed graduated cylinder’s jacket, respectively, using rubber tubing.

➁ Switch on the super-thermostatic water bath, set the experimental temperature to 45 ± 0.5 °C, and start the water circulation to preheat and stabilize the temperature of the jacketed graduated cylinder.

➂ Using a separatory funnel, add the test solution to the jacketed graduated cylinder up to the 50 mL mark. Whilst adding the solution, to prevent foaming, ensure that the lower end of the separatory funnel’s measuring tube is in contact with the wall of the jacketed graduated cylinder, allowing the solution to flow down the wall.

➃ Using a homemade tapered funnel, pour part of the test solution into the separatory funnel up to the 150 mm mark; then, using a measuring cylinder, slowly pour 50 mL of the test solution into the separatory funnel.

➄ Adjust the apparatus so that the axis (center line) of the jacketed graduated cylinder aligns with the axis of the separating funnel’s measuring tube, and ensure that the lower end of the measuring tube is positioned at the 250 mm mark relative to the liquid level of the 50 mL solution inside the graduated cylinder.

➅ Turn the separatory funnel knob to allow the test solution to flow continuously until the liquid level drops to the 150 mm mark; record the result Vmax when the flow stops, then measure and record the volume of foam (foam only) in the graduated cylinder at 30-s intervals.

➆ Repeat steps ➁ to ➅ twice, calculate the average foam volume, provided that the absolute difference between the two independent test results obtained under repeatable conditions does not exceed 5 per cent.

➇ Data processing: record the result Vmax as the foaming volume of the surfactant solution, which characterizes the foaming capacity of the surfactant. The specific calculation formula is shown in Equation (1).

(1) $$FCI=\frac{3}{4}V_{\max}t_{1/2}$$

In the equation: *FCI*—Foam Complexity Index, (mL·min); *V_max_*—Maximum foam volume, (mL); *t*_1/2_—Half-life, (min).

(b) Oil–water interfacial tension testing:

➀ Switch on the surface tension meter, allow the instrument to warm up, and set the experimental temperature to 45 °C.

➁ Rinse the measuring tube three times with distilled water, then rinse it once with a surfactant solution, and finally fill the measuring tube with the surfactant solution to be tested.

➂ Draw up the oil sample using a micropipette and dispense it into the measuring tube to form a droplet of suitable size; remove any air bubbles from the measuring tube, then place the measuring tube into the rotating cylinder and preheat for 5 min.

➃ Set the rotational speed to 3000 rpm and adjust the camera to observe the position of the oil droplet.

➄ Start the interfacial tension meter, measure the initial height and length of the oil droplet, and calculate the interfacial tension (calculated automatically by the computer); set the camera to take photographs at 5-min intervals, measure the height and length of the oil droplet at different time points (when the length-to-height ratio exceeds 4, measure only the height), calculate the interfacial tension, and record this as the dynamic interfacial tension.

➅ Measure the oil droplet at three consecutive time intervals; if the dimensional error between measurements is <0.1, record this as the steady-state surface tension value. Plot the measured dynamic surface tension against the test time; the lowest point is recorded as the instantaneous minimum surface tension. The specific calculation formula is shown in Equation (2).

(2) $$\sigma =\frac{1.2336∆\rho \;f(Z/Y){\left(Y/n\right)}^{3}}{P^{2}}$$

In the equation: σ—oil–water interfacial tension, mN/m; ∆ρ—density difference between the high-density and low-density phases, g/cm^3^; *Y*—diameter of the oil column, 10^−4^ m; *P*—reciprocal of the rotational speed, ms/r; n—refractive index of the aqueous phase; *Z*—length of the oil column, 10^−4^ m; *f*(*Z/Y*)—correction factor.

#### 2.2.2. Evaluation of the Plugging Performance of the LIFT-NSF Foam System

(1) Inject water at a constant rate until the pressure at the inlet and outlet ends stabilizes, record the pressure difference between the two ends, and calculate the initial liquid-phase permeability of the core, *K_w_*_1_.

(2) Mix nitrogen and LIFT-NSF foam concentrate at the optimal gas-to-liquid ratio and inject the mixture into the core at a constant rate. Record the pressure difference between the inlet and outlet ends, and calculate the average liquid-phase permeability, *K_L_*.

(3) After injecting the target plugging dose, inject formation water at a constant rate until the pressure at the inlet and outlet ends stabilizes. Record the pressure difference between the two ends and calculate the liquid-phase permeability (*K_w_*_2_) of the subsequent water drive in the core.

(4) Once the displacement process is fully complete, calculate the post-displacement resistance coefficient (*R_f_*) and the residual resistance coefficient (*R_Rf_*), and compute *R_f_* and *R_Rf_* separately according to Equations (3) and (4).

(3) $$R_{f}=\frac{\mu_{w}K_{W1}}{\mu_{f}K_{W2}}$$

(4) $$R_{Rf}=\frac{K_{W1}}{K_{W2}}$$

In the equation: $\mu_{f}$—Viscosity of the foam solution, mPa·s; $\mu_{w}$—Viscosity of formation water, mPa·s; $K_{W1}$—Effective permeability of the initial aqueous phase, mD; $K_{W2}$—Effective permeability of the water-driven aqueous phase, mD.

#### 2.2.3. Optimization of Injection Parameters

(1) The core was evacuated for 12 h, saturated with formation water, and the pore volume of the core was calculated.

(2) Oil-displacement water: Displace water from the core using 3–5 PV of formation oil, bringing the oil saturation in the core close to the actual initial oil saturation of the reservoir, and calculate the bound water saturation.

(3) Water-displacement oil: Inject water at a constant flow rate of 0.1 mL/min, measuring oil production, water production, and pressure changes at the outlet.

(4) Foam-based oil displacement: Foam is generated by alternately injecting N_2_ and LIFT-NSF foam concentrate at constant pressure. Tests are conducted under constant pressure conditions (4 MPa, 5 MPa, 6 MPa), with plug volumes of 0.15 PV, 0.2 PV, 0.3 PV, 0.6 PV, and gas-to-liquid ratios of 1:1, 2:1, 3:1, 4:1) into the core, and measure oil production, water production, and pressure changes at the outlet;

(5) Follow-up water flooding: Inject water at a constant rate of 0.1 mL/min, and measure oil production, water production, and pressure changes at the outlet.

#### 2.2.4. Oil Displacement Using Heterogeneous Core Samples

(1) Sort the core samples by air permeability, then place the low-permeability and high-permeability cores in sequence into a long-core holder configured in parallel. Evacuate the system and saturate it with formation water.

(2) Oil displacement: Displace the water in the core with formation oil at 3–5 PV until no water flows out of the core outlet, thereby establishing confined water.

(3) Water-in-oil displacement: Inject water at a constant rate of 0.1 mL/min, measuring oil production, water production, and pressure changes at the outlet.

(4) Foam-based oil displacement: Generate foam by alternately injecting N_2_ and LIFT-NSF foam concentrate at constant pressure. Inject into the core at different pressures, with varying plug dosages and gas-to-liquid ratios, while measuring oil production, water production, and pressure changes at the outlet.

(5) Follow-up water flooding: Inject water at a constant rate of 0.1 mL/min, and measure oil production, water production, and pressure changes at the outlet.

## 3. Results and Discussion

### 3.1. Evaluation of the LIFT-NSF Foam System

As shown in Table 3, The results of foam performance and surface tension tests on different formulation systems indicate that, when used alone, LS-12 exhibits superior overall foaming and foam-stabilizing performance to LT-14, whilst LT-14 is more effective at reducing surface tension. A clear synergistic effect is observed when LS-12 and LT-14 are blended in a binary system, with significant improvements in foam volume, foam half-life, and the comprehensive foam index, alongside a substantial reduction in surface tension. Upon the introduction of 0.03% WB additive into this binary blend system, the foam half-life was further extended to 100 min, the surface tension was reduced to a minimum of 6.46 × 10^−2^ mN/m, and the foam comprehensive index reached 15,000 mL·min, resulting in the optimal overall foaming and foam-stabilizing performance. The overall trend indicates that the lower the interfacial tension of the system, the greater the foam stability; the ternary blend of 0.4% LS-12 + 0.1% LT-14 + 0.03% WB was identified as the optimal foam-driving formulation in this experiment.

**Figure 1:**
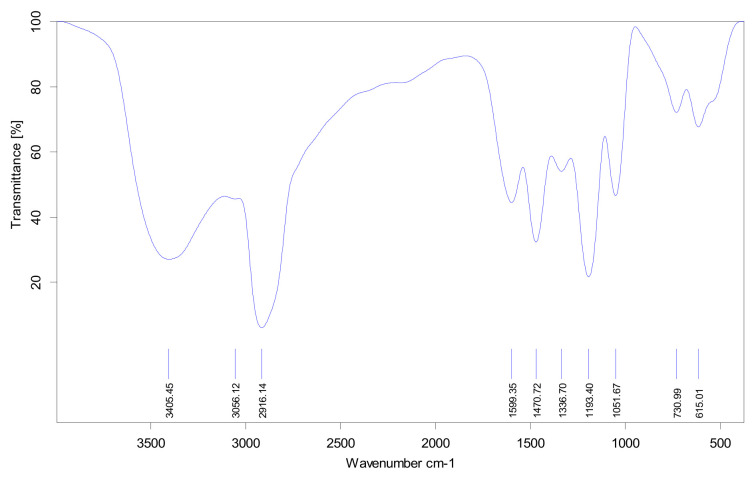
Infrared spectrum of a di-dodecyl hydroxysulphate surfactant.

**Figure 2:**
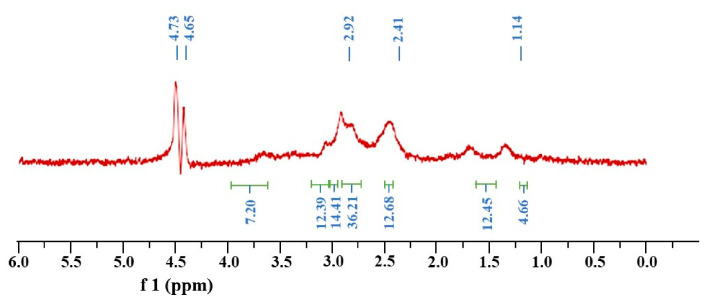
^1^H NMR spectrum of a di-12-alkyl hydroxysulphate surfactant.

As shown in Figure 1 and Figure 2, the characteristic absorption peak at 3405.45 cm^−1^ corresponds to the stretching vibration of the -OH- group; the peak at 1470.72 cm^−1^ corresponds to the bending vibration of the C-N bond; the peak at 1599.36 cm^−1^ corresponds to the in-plane bending-stretching vibration of the -CH- group; at 3056.12 cm^−1^ is the out-of-plane bending vibration peak of the -NH- group; at 1193.40 cm^−1^ and 1051.67 cm^−1^ are the characteristic absorption peaks of the antisymmetric and symmetric stretching vibrations of the -SO_3_ group; at 2916.14 cm^−1^ is the characteristic absorption peak of the stretching vibration of methyl and methylene groups; 730.99 cm^−1^ corresponds to the long-chain methylene group in the molecule. It can be seen that the synthetic product contains hydroxyl (-OH) and sulphonic acid (-SO_3_) groups, as well as -NH- groups linking hydrophobic carbon chains and C-N groups linking two hydrophobic chains. This is consistent with the molecular structure of the target compound LS-12.

Accordingly, a surfactant with a hydrophilic head and a hydrophobic chain, which exhibits the best foaming properties, will be selected as the salt-resistant foaming agent. It not only possesses good foaming properties but also demonstrates relatively outstanding foam stability.

### 3.2. Discussion on Foam Stabilization Mechanism Under High-Salinity Conditions

The LIFT-NSF foam system operates in a formation water environment with a total salinity of 118,212 mg/L. Under such extreme ionic strength conditions, the electrical double layer of the carboxyl-modified silica nano-particles collapses due to severe Debye shielding, rendering electrostatic repulsion negligible. Consequently, the stability of the foam thin liquid films in this system cannot rely on electrostatic mechanisms and must depend entirely on other forces.

The dominant stabilization mechanism governing the LIFT-NSF foam is Pickering steric stabilization, provided by the irreversibly adsorbed carboxyl-modified hydrophilic nano-SiO_2_ particles (0.03% WB) at the gas–liquid interface. The contact angle of these nano-particles at the gas–liquid interface is a critical parameter determining their adsorption strength. Our static imbibition experiments measured the water contact angle to be 17.0°, indicating that the carboxyl-modified nano-particles exhibit moderate hydrophilicity (as shown in Table 4). This contact angle ensures that the nano-particles are strongly held at the interface while remaining well-dispersed in the aqueous phase, which is crucial for their transport through the porous medium.

To quantitatively substantiate the stability of our foam system, we calculated the desorption free energy barrier for a single nano-particle at the gas–liquid interface (Equation (5)). The desorption energy is given by the well-established equation:

(5) $$\Delta G_{des}=\pi r^{2}\Upsilon {\left(1-|\cos\theta_{w}|\right)}^{2}$$

Based on our system parameters—particle radius (*r*) in the range of 10–20 nm, liquid–gas surface tension (*γ*) of 6.46 × 10^−2^ mN/m, and contact angle (*θ_w_*) of 17.0—the desorption free energy barrier for a single nano-particle is calculated to be several orders of magnitude greater than the thermal energy. This enormous energy barrier means that once the nano-particles adsorb at the gas–liquid interface, they are effectively irreversibly attached. This irreversibility is the fundamental reason why the LIFT-NSF foam can resist the high shear forces encountered within tight pore throats during core flooding, preventing bubble coalescence and maintaining foam stability.

This theoretical framework is directly supported by our experimental observations. In our core flooding experiments, stable foam could be generated even in high-permeability channels subject to high local shear rates, as evidenced by the high residual resistance coefficient (*R_Rf_* = 4.013) observed in the 5.158 mD core.

As shown in Table 5, the irreversibly adsorbed nano-particles create a rigid, steric barrier around the gas bubbles, which prevents the drainage of the liquid film between two approaching bubbles, effectively suppressing coalescence. This is in stark contrast to surfactant-only foams, which would rapidly destabilize under such high-shear conditions in high-salinity brine. The enhanced foam stability resulting from this Pickering mechanism is what gives the LIFT-NSF system its excellent selective plugging capability, as demonstrated by the resistance coefficient increasing from 1.086 to 15.468 with increasing core permeability from 0.505 mD to 5.158 mD.

In summary, the foam stability of the LIFT-NSF system under extreme salinity is governed by Pickering steric stabilization rather than electrostatic repulsion. The moderate hydrophilicity (17.0°) of the carboxyl-modified silica nano-particles provides a sufficiently high desorption free energy barrier to prevent bubble coalescence under reservoir shear conditions, thereby ensuring the system’s excellent plugging and oil displacement performance.

### 3.3. Evaluation of LIFT-NSF Foam Plugging and Injection Performance

#### 3.3.1. Result of Plugging and Injection Tests

**Figure 3:**
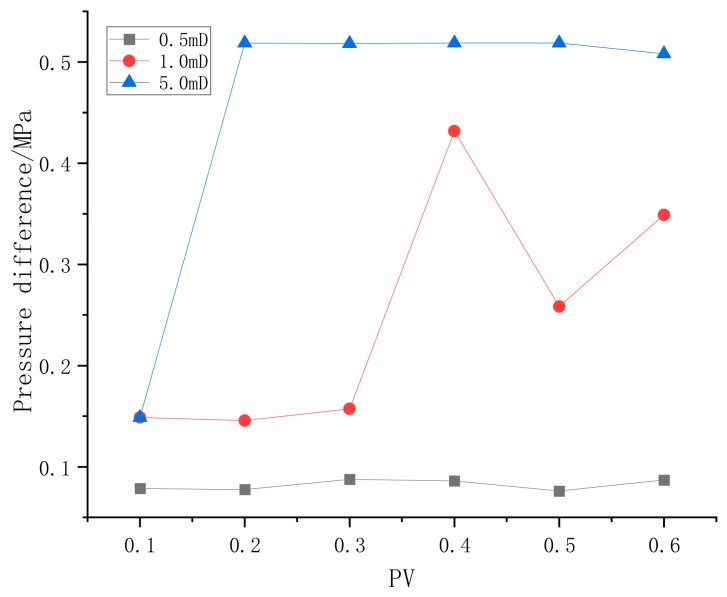
Evaluation of the injection performance of the LIFT-NSF foam system.

Three rock cores with different permeability values were selected from the target reservoir. Under conditions of constant pressure (5 MPa), a single-stage plug volume of 0.2 PV (0.1 PV liquid and 0.1 PV gas, with a total injection volume of 0.6 PV), and a gas-to-liquid ratio of 1:1, the plugging and injection performance of the LIFT-NSF foam system on the rock cores was investigated. The results are shown in Table 5 and Figure 3.

Table 5 presents the results of the plugging performance of the LIFT-NSF foam system on rock cores with different permeability values. As shown in Table 5, the LIFT-NSF foam system exhibits selective plugging capability for different permeable layers; the plugging strength is positively correlated with the permeability of the rock core, enabling effective fluid flow diversion and improving sweep efficiency in heterogeneous reservoirs. The evaluation results of the plugging performance indicate that the resistance coefficient of the LIFT-NSF foam system increases significantly with rising core permeability; in a core with a permeability of 5.158 mD, the resistance coefficient reaches 15.468, while the residual resistance coefficient remains at 4.013, demonstrating that the system possesses a potent and sustained plugging capability for high-permeability layers. In contrast, for a low-permeability core of 0.505 mD, the resistance coefficient is only 1.086, with a weak blocking effect, demonstrating good permeability-gradient-selective blocking characteristics, which is conducive to achieving fluid flow reorientation and expanding the swept volume in heterogeneous low-permeability reservoirs.

Figure 3 shows the evaluation of the injection performance of the LIFT-NSF foam system for cores with different permeabilities. As shown in Figure 1, the injection pressure differential is positively correlated with core permeability; the higher the core permeability, the greater the injection pressure differential. Throughout the entire injection process, the injection pressure differential fluctuated within the range of 0.08–0.52 MPa, with the maximum differential reaching only 0.52 MPa, indicating that the LIFT-NSF foam system can be successfully injected into low-permeability, dense cores or reservoirs.

In summary, the selected LIFT-NSF foam system can provide a certain degree of plugging effect for low-permeability cores (0.505–5.158 mD) while maintaining good injectability.

#### 3.3.2. Non-Newtonian Foam Transport and Selective Plugging Mechanism in Porous Media

The flow of foam within porous media deviates significantly from classical Newtonian mechanics. Foam fluids exhibit distinct yield stress and shear-thinning (pseudoplastic) behavior, where the apparent viscosity decreases with increasing shear rate. This non-Newtonian rheology is the fundamental physical basis governing the selective plugging behavior observed in heterogeneous reservoirs.

As shown in Table 5, the mechanism of selective plugging in high-permeability channels is intrinsically linked to the local shear rate. The rate of foam generation in porous media is directly proportional to the local shear rate. In high-permeability channels, the higher flow velocity and larger pore throats create elevated local shear rates, which promote more vigorous in situ foam generation. This results in a marked reduction in gas mobility within these highly swept zones. Conversely, in low-permeability zones, the limited pore space and lower flow velocities generate minimal shear, leading to negligible foam generation and minimal mobility reduction. This mechanism is directly supported by our experimental data: the resistance coefficient of the LIFT-NSF foam system increases significantly with rising core permeability—from 1.086 at 0.505 mD to 15.468 at 5.158 mD. In the 0.505 mD core, the minimal foam generation results in a weak blocking effect (resistance coefficient of 1.086), which is beneficial for maintaining injectivity in low-permeability layers.

As foam accumulates and restricts mobility in the high-permeability zones, the local capillary pressure increases. This elevated capillary pressure effectively diverts the subsequently injected water phase into adjacent unswept, low-permeability matrices. This mechanism is vividly demonstrated by our heterogeneous core flooding results: during initial water flooding, the recovery rate in low-permeability tube cores remained at 0%, indicating that water preferentially channeled through high-permeability pathways (Please refer to Section 3.5 for detailed experimental data.).

However, after the injection of the LIFT-NSF foam system, the subsequent water flooding achieved recovery rates of up to 6.54% in low-permeability layers (when the permeability gradient was 10), confirming the successful diversion of the displacing fluid.

To provide a rigorous theoretical foundation for these observations, we have incorporated an extended form of Darcy’s Law that accounts for the non-Newtonian rheology of foam flow. For a shear-thinning fluid, the apparent viscosity (*μ_eff_*) can be described by the power-law (Ostwald-de Waele) model (Equations (6) and (7)):

(6) $$\mu_{eff}=K\cdot \Upsilon^{n-1}$$

where *K* is the consistency index, *γ* is the shear rate, and n is the flow behavior index (*n* < 1 for shear-thinning fluids). Substituting this into the extended Darcy’s Law for multiphase flow yields:

(7) $$V_{f}=-\frac{K_{rf}\cdot K}{\mu_{eff}}\cdot △P=-\frac{K_{rf}\cdot K}{K\cdot \Upsilon^{n-1}}\cdot △P$$

This formulation demonstrates that in high-permeability zones where shear rates are elevated, the decrease in apparent viscosity (due to shear-thinning) is counteracted by the massive increase in foam generation rate. The net effect is a substantial reduction in foam mobility, which increases the flow resistance and promotes fluid diversion into low-permeability zones. This extended Darcy’s Law provides a rigorous scientific underpinning for our experimental interpretations and explains why the LIFT-NSF foam system can effectively seal high-permeability reservoirs with permeability gradients below 50, while maintaining sufficient injectivity in low-permeability layers to achieve a comprehensive recovery enhancement of over 10% in heterogeneous oil reservoirs.

### 3.4. Optimization of Injection Parameters

#### 3.4.1. Injection Pressure

To determine the optimal injection rate for the LIFT-NSF foam system, three rock cores with permeabilities of approximately 1 mD were selected for this experiment to match the typical characteristics of the target reservoir, which is characterized by low permeability and low porosity. At the same time, considering the high viscosity of the foam fluid itself, which could cause a rapid increase in injection pressure differential during injection, as well as subsequent issues such as failure to inject due to excessively high pressure differentials during water flooding, a constant pressure differential displacement method was selected. This approach allows for effective control of the injection pressure differential and displacement fluid injection, while reasonably controlling the foam fluid injection rate through constant pressure differential variations.

**Figure 4:**
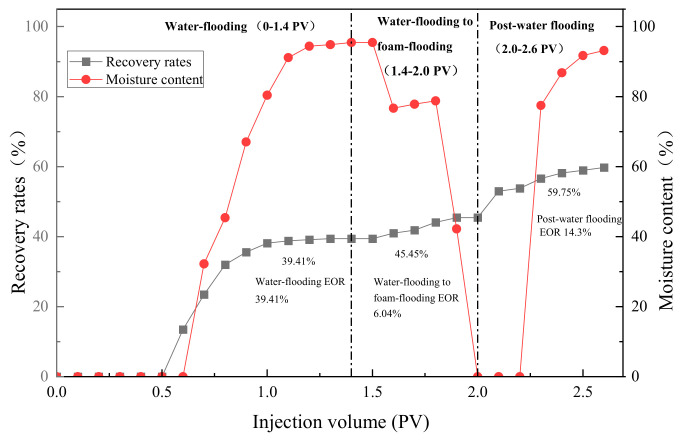
Effect of a 4 MPa injection pressure differential on oil displacement.

**Figure 5:**
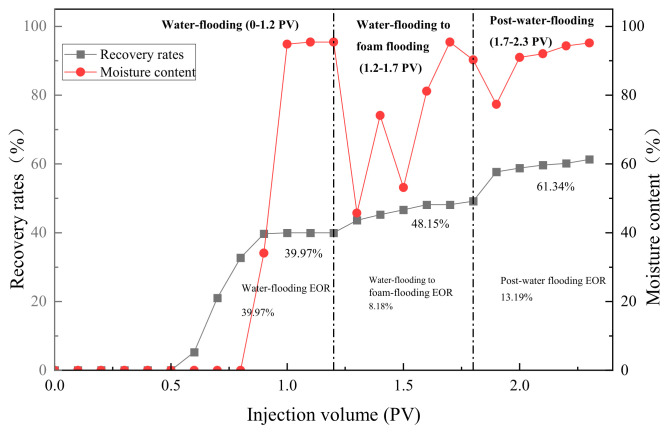
Effect of a 5 MPa injection pressure differential on oil displacement.

**Figure 6:**
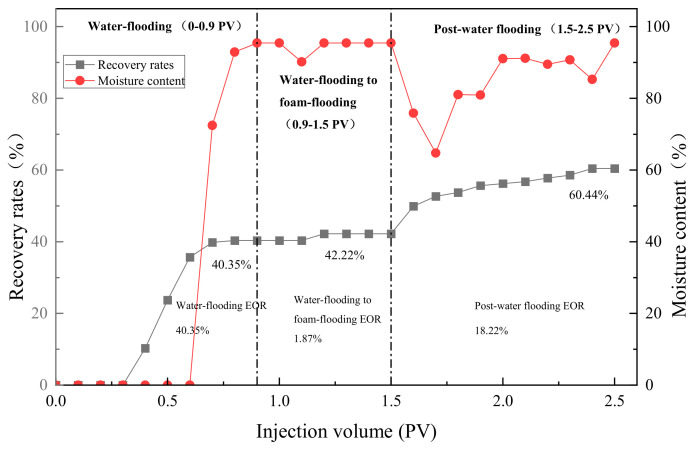
Effect of a 6 MPa injection pressure differential on oil displacement.

The specific experimental conditions were as follows: a gas-to-liquid ratio of 1:1, injection pressure differentials of 4 MPa, 5 MPa, and 6 MPa, a foam (liquid + gas) plug of 0.2 PV, a total injection plug of 0.6 PV, and three cycles of alternating gas and liquid injection. The effects of different pressure differentials on the oil recovery performance of the LIFT-NSF foam system are shown in Table 6 and Figure 4, Figure 5 and Figure 6.

As shown in Table 6 and Figure 4, Figure 5 and Figure 6, as the injection pressure difference increases, the increase in crude oil recovery rate due to foam flooding initially rises and then decreases. Specifically, at an injection pressure difference of 5 MPa, foam flooding can increase the crude oil recovery rate by up to 8.18%, whereas at an injection pressure difference of 6 MPa, the increase is only 1.87%. Analysis suggests that this may be due to an excessively high injection pressure differential, causing the foam solution and gas to advance rapidly through the large channels in the rock core without effectively forming foam and mobilizing residual oil. Consequently, the ultimate increase in crude oil recovery is relatively small.

For the subsequent water flooding, the crude oil recovery rate exhibits a trend of first decreasing and then increasing as the injection pressure differential increases. Specifically, when the injection pressure difference was 6 MPa, the subsequent water drive increased the crude oil recovery rate by up to 18.23%. Analysis suggests this may be because, during the water drive, the rapidly advancing foam liquid and deoxygenated air began to form bubbles. This facilitated an expanded reach of the subsequent injected water and enhanced the stripping of residual oil by the foamed fluid, thereby improving the crude oil recovery rate during the subsequent water drive.

Regarding the overall improvement in waterflood recovery, as the injection pressure differential increases, the total recovery rate first increases and then decreases. When the injection pressure differential is 5 MPa, the LIFT-NSF foam system improves recovery by 21.37% compared to conventional waterflooding, demonstrating the best oil displacement performance. It was also observed that, for cores with similar permeability, the injection rate decreased significantly as the injection pressure differential increased. This fully demonstrates that the LIFT-NSF foam system exhibits the characteristic of blocking large pores but not small ones, making it well-suited for low-permeability reservoirs.

In summary, subsequent experiments will optimize the parameters for the foam drive at a constant pressure of 5 MPa.

#### 3.4.2. Foam Plug Volume

**Figure 7:**
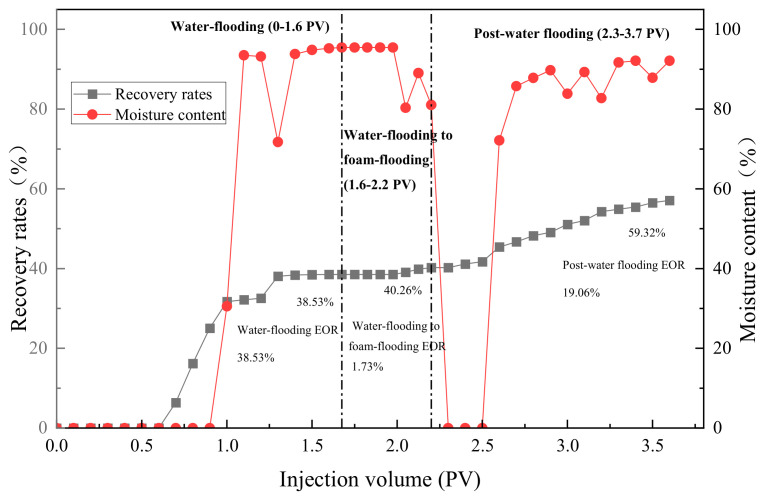
Effect of a plug volume of 0.15 PV on oil displacement.

**Figure 8:**
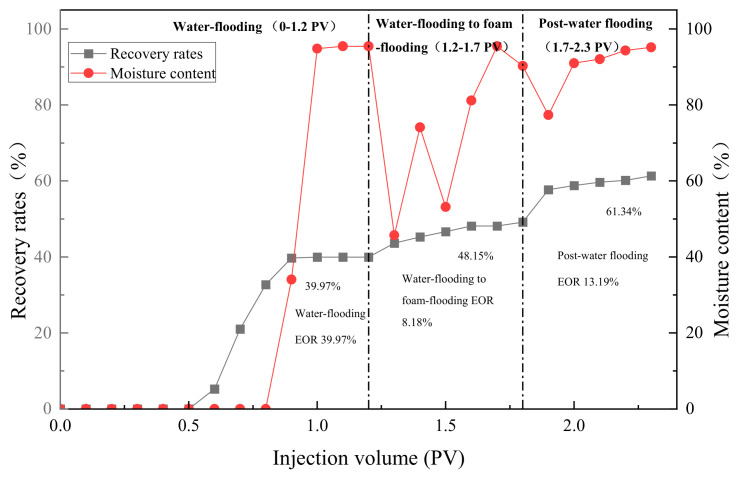
Effect of a plug volume of 0.2 PV on oil displacement.

**Figure 9:**
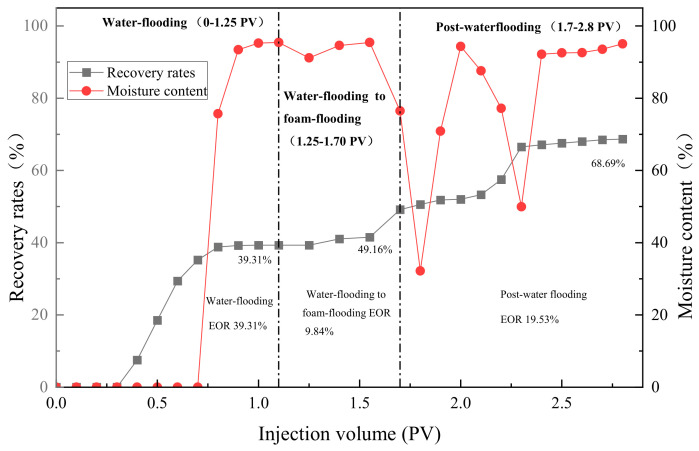
Effect of a plug volume of 0.3 PV on oil displacement.

**Figure 10:**
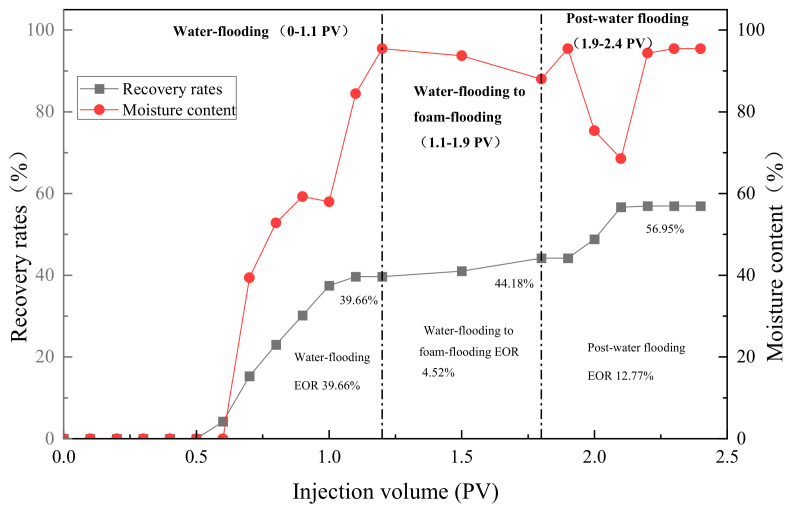
Effect of a plug volume of 0.6 PV on oil displacement.

Under conditions of an injection pressure difference of 5 MPa, a total injection volume of 0.6 PV, and a gas-to-liquid ratio of 1:1, the effect of foam plug volume (liquid + gas) (0.15 PV, 0.2 PV, 0.3 PV, 0.6 PV) on the crude oil recovery efficiency of the LIFT-NSF foam flooding method was investigated. The results are shown in Table 7 and Figure 7, Figure 8, Figure 9 and Figure 10.

Table 7 and Figure 7, Figure 8, Figure 9 and Figure 10 show that as the number of injection cycles of N_2_ and LIFT-NSF foam solution decreases, and as the foam plug volume per cycle increases, the subsequent water drive following the foam drive exhibits a trend of first increasing and then decreasing in its contribution to the total crude oil recovery rate. Specifically, when the foam plug volume per cycle was 0.3 PV and the number of alternating cycles was two, the foam drive increased the total recovery rate of the subsequent water drive by 29.37%, yielding the best results; when the foam plug volume per cycle was at its maximum (0.6 PV) and the number of alternating gas–liquid injection cycles was one, the foam drive increased the total recovery rate of the subsequent water drive by 17.29%, yielding the poorest results.

The primary reason is that the probability, volume, or extent of foam formation when the injected N_2_ comes into contact with the foaming liquid within the core pores is limited, resulting in a relatively small amount of foam. Additionally, partial defoaming occurs upon contact with oil, preventing the formation of an effective foam plug. Consequently, the foam drive has a limited effect on increasing the oil displacement volume, leading to a low total recovery rate. Conversely, appropriately reducing the volume of a single gas-liquid plug can effectively increase the frequency of gas-liquid alternation or contact, which is more conducive to the effective formation and stability of foam, thereby fully leveraging the effectiveness of foam flooding in enhancing water flooding recovery.

Based on this, subsequent experiments will continue to optimize foam flooding parameters using a constant pressure difference of 5 MPa and a single gas–liquid plug volume of 0.3 PV.

#### 3.4.3. Gas-to-Liquid Ratio

**Figure 11:**
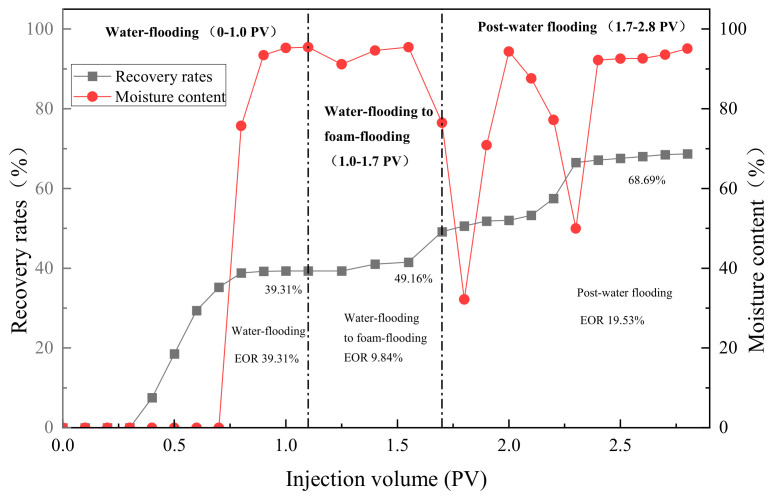
Effect of a 1:1 gas-to-liquid ratio on oil displacement.

**Figure 12:**
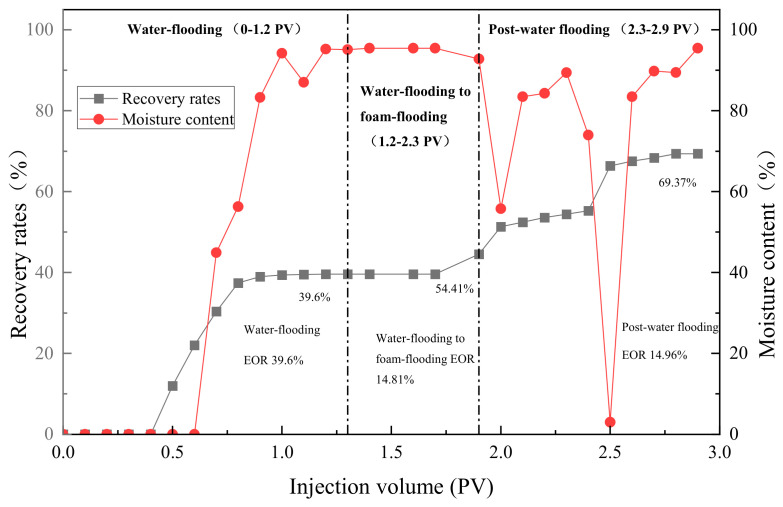
Effect of a 2:1 gas-to-liquid ratio on oil displacement.

**Figure 13:**
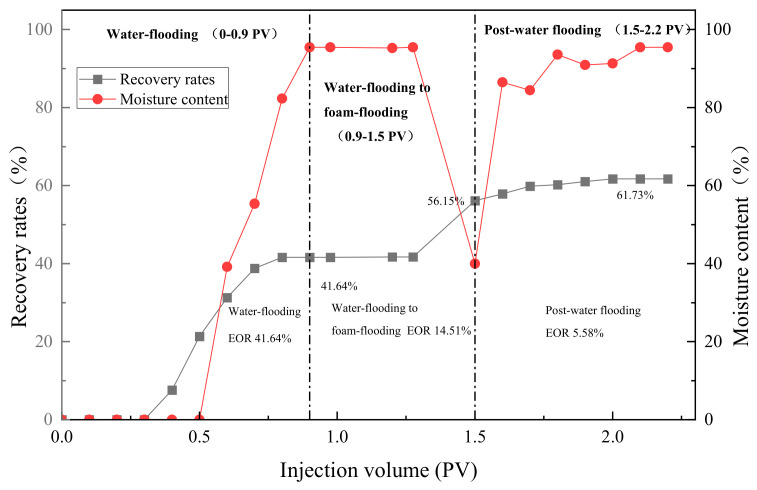
Effect of a 3:1 gas-to-liquid ratio on oil displacement.

**Figure 14:**
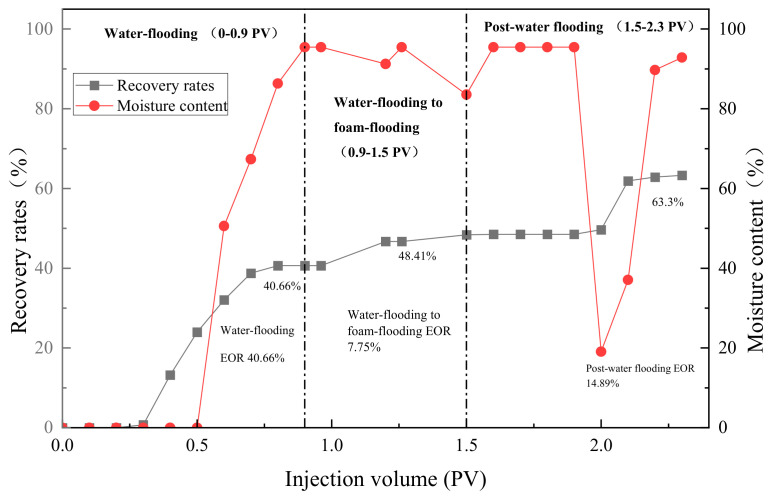
Effect of a 4:1 gas-to-liquid ratio on oil displacement.

Under conditions of an injection pressure differential of 5 MPa, a total plug volume of 0.6 PV, a single-cycle gas-to-liquid plug volume of 0.3 PV, and two cycles of alternating gas and liquid injection, the effect of varying injection gas-to-liquid ratios (1:1, 2:1, 3:1, 4:1) on the LIFT-NSF foam oil displacement performance was investigated. The results are shown in Figure 11, Figure 12, Figure 13 and Figure 14.

Figure 12, Figure 13 and Figure 14 show that different gas-to-liquid ratios have a significant impact on enhancing crude oil recovery. Following water flooding, the recovery rate from foam flooding initially increases and then decreases as the gas-to-liquid ratio rises. Analysis reveals that appropriately increasing the gas-to-liquid ratio promotes contact between the gas and liquid phases, forming relatively continuous foam that blocks large pores within the rock matrix, thereby increasing the displacement volume of the water phase during subsequent water flooding. Conversely, when the N_2_ injection volume or gas-to-liquid ratio is too high, it tends to reduce the amount of foam generated or even result in a continuous gas phase, similar to gas flooding, ultimately leading to an insignificant improvement in recovery rate. From the perspective of overall crude oil recovery, when the gas-to-liquid ratio is 2:1, the subsequent water-flooding recovery rate can reach a maximum of 29.77%. Therefore, the optimal gas-to-liquid ratio is determined to be 2:1.

Based on the comprehensive parameter optimization study described above, the optimal injection parameters for the LIFT-NSF foam system are a constant pressure of 5 MPa, a gas-liquid plug volume of 0.3 PV, and a gas-liquid ratio of 2:1. Consequently, all subsequent LIFT-NSF foam flooding experiments were conducted under these parameters.

### 3.5. Oil Recovery Performance in Heterogeneous Formations

Using the injection rate, packer volume, and gas-to-liquid ratio optimized in Section Optimization of Injection Parameters, N_2_ foam recovery experiments using the LIFT-NSF foam system were conducted in parallel high- permeability and low-permeability rock cores with different permeability gradients to investigate the effect of formation heterogeneity on the oil recovery performance of the LIFT-NSF foam system. The results are shown in Table 8.

As shown in Table 8, the recovery rate in water flooding is primarily influenced by permeability and permeability gradients. During water flooding, the recovery rate in high-permeability tube cores depends mainly on the core’s permeability; the higher the permeability, the greater the recovery. This also indicates that during water flooding, water preferentially enters high-permeability channels and displaces crude oil.

In contrast, the recovery rate in low-permeability tube cores remained at 0 throughout the entire water flooding process. Analysis of the causes reveals that this is primarily due to the presence of permeability gradients, which make it easier for the displacing water phase to pass through high-permeability channels, while the mobilization of crude oil in low-permeability channels is significantly reduced. Therefore, it is necessary to plug the high-permeability channels after water flooding to force water to gradually enter the low-permeability channels during subsequent water flooding operations, thereby increasing the water flooding recovery rate in the low-permeability layers.

After injecting 0.4 PV of LIFT-NSF foam concentrate and conducting subsequent water flooding, the recovery rates for both high-permeability and low-permeability rock cores increased. When the permeability gradient was 10, the recovery rates increased by 13.38% and 6.54%, respectively; when the permeability gradient is 35, the increases in recovery rates are 11.69% and 2.14%, respectively; when the permeability gradient is 50, the increases are 9.33% and 1.76%, respectively, and the rate of increase has begun to decline compared to when the permeability gradient is 10 or 35.

When the permeability gradient was 60, the pores and throat channels in the high-permeability core were well developed. Compared to the low-permeability core (permeability of 1.0 mD), the permeability of the high-permeability core had reached the medium-to-high permeability range. During foam flooding at this stage, the foam solution still primarily enters the high-permeability tube channels, so the resulting foam cannot effectively seal these pathways. Consequently, when water flooding is subsequently resumed, the injected water continues to flow along the established dominant flow paths, resulting in a 0% increase in recovery rate for the low-permeability tube core.

When the permeability gradient is 50 and 60, the total recovery rate still increases by 6.62% and 19.24%, respectively. Analysis of the reasons indicates that this is primarily because, during foam flooding, the foam preferentially enters large throat channels and pores, creating a certain degree of blocking effect during this process; simultaneously, the foam also enters small pores and throat channels, thereby carrying out a portion of the residual oil. During the subsequent water flooding phase, because the foam has blocked the larger channels and pores, the injected water can reach more small pores and channels, thereby improving the sweep efficiency of the water phase and ultimately increasing the total recovery rate.

In summary, the LIFT-NSF foam system can provide effective sealing in highly mineralized environments (mineralization > 110,000 mg/L) and high-permeability reservoirs with a permeability gradient below 50, and can increase the total recovery rate of heterogeneous reservoirs by up to 10% or more.

### 3.6. Static Imbibition Oil Recovery Performance

**Figure 15:**
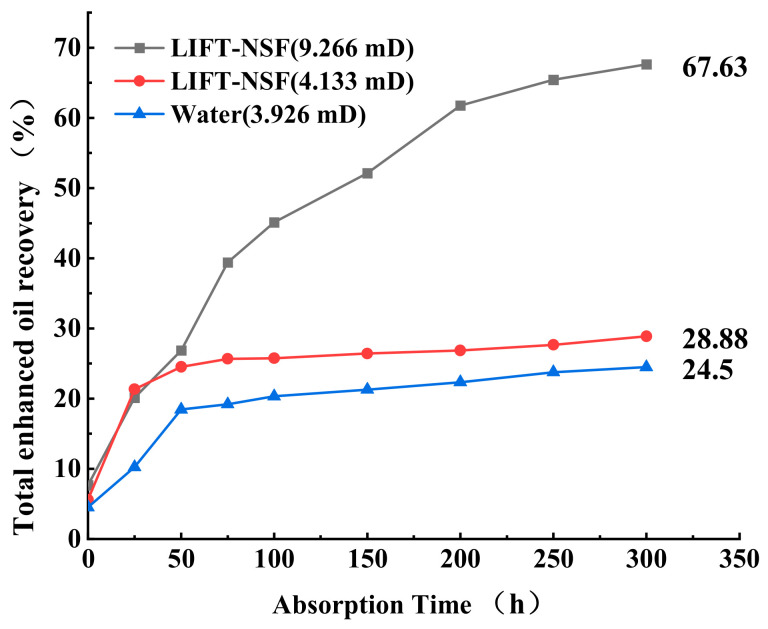
Comparison of static imbibition performance of various fluids at different permeability rates.

Core samples with different permeabilities were placed in formation water and LIFT-NSF foam solutions to test the static imbibition oil recovery performance of different systems on core samples with varying permeabilities from the target reservoir. The results are shown Figure 15.

Figure 15 presents a comparison of the static imbibition and oil displacement effects for cores with different permeabilities in various fluids. As shown in Figure 15:

(1) As the percolation time increases, both formation water and the LIFT-NSF foam system are capable of displacing crude oil from the rock core, and the oil displacement efficiency of the LIFT-NSF foam system consistently outperforms that of formation water. When the core permeability is around 4.0 mD, the static imbibition recovery rate of LIFT-NSF is 4.38% higher than that of formation water, indicating that the LIFT-NSF foam system possesses a certain degree of permeability enhancement. Analysis of the reasons reveals that the Microscopic Displacement Enhancement (Imbibition Effect) and the Macroscopic Sweep Efficiency Improvement (Selective Plugging Effect). Firstly, the moderate reduction in interfacial tension reduces the capillary pressure barrier (*P_c_* = 2*σcosθ*/*r*) within the fine pore throats of the reservoir rock. This allows the LIFT-NSF system to more effectively imbibe into the smallest pores and displace trapped oil. Our static imbibition experiments directly support this, showing that the LIFT-NSF system can enhance oil recovery by up to 4.38% compared to pure formation water, even in low-permeability (4.0 mD) cores. Additionally, the system was observed to reduce the water contact angle from 39.6° to 17.0°, further enhancing the capillary-driven imbibition process. Secondly, in situ foam generation drastically increases the apparent viscosity of the displacing phase. This viscous foam preferentially enters and plugs high-permeability thief zones, diverting subsequent injection water into the previously unswept low-permeability layers. Our experiments demonstrate the efficacy of this mechanism: for cores with a permeability gradient below 50, the foam system can increase the recovery rate of low-permeability layers from 0% to over 10%, thereby significantly enhancing the macroscopic sweep efficiency.

(2) When the rock core permeability was 9.266 mD, the static imbibition recovery rate of the LIFT-NSF foam solution increased significantly with increasing permeation time, with the crude oil recovery rate reaching a maximum of 67.63%. This is because the nano-particles in LIFT-NSF alter rock wettability, enhancing hydrophilic properties and facilitating the desorption of oil from pore surfaces, thereby further improving recovery rates.

In summary, the developed LIFT-NSF foam system is suitable for application in low-permeability reservoirs. During the oil displacement process, as the permeability of the reservoir core continues to increase, the developed LIFT-NSF foam system, compared to formation water without surfactants, can to some extent alter the contact angle of the reservoir rock and enhance its hydrophilicity. This transforms capillary forces from a source of flow resistance into a driving force for oil displacement, ultimately improving displacement efficiency.

## 4. Conclusions

(1) A new technology for enhancing recovery rates in low-permeability reservoirs was proposed using a low-surface-tension, salt-tolerant nano-particle-stabilized foam system, LIFT-NSF (0.4% LS-12 + 0.1% LT-14 + 0.03% WB). When mixed with nitrogen to form, this system exhibits excellent injectability and plugging performance, with a resistance coefficient that increases (1.09–15.47) as permeability increases (0.5–5 mD).
(2) The optimal injection parameters for the LIFT-NSF foam system are a constant pressure of 5.0 MPa, a plug volume of 0.3 PV, and a gas-to-liquid ratio of 2:1. Under these optimal conditions, the system can increase the recovery rate of subsequent water flooding by up to 29.77%, demonstrating significant effectiveness.
(3) The LIFT-NSF foam system can effectively seal high-permeability reservoirs in high-salinity environments with permeability gradients below 50. It also has the ability to activate low-permeability layers (with permeabilities around 1 mD) and can increase the total recovery rate of heterogeneous reservoirs by up to 10% or more.
(4) The LIFT-NSF foam system can effectively penetrate the small pores and throat channels in low-permeability rock cores and displace crude oil through capillary forces. Compared to pure water without surfactants, this system demonstrates superior permeation-enhancing and oil-displacement effects.

## Figures and Tables

**Table 1:** Core physical properties.

Core No.	Average Length (cm)	Average Diameter (cm)	Air Permeability (mD)
1	8.05	2.43	1.004
2	8.09	2.48	1.004
3	8.05	2.47	1.143
4	7.67	2.45	0.505
5	7.66	2.46	1.003
6	7.68	2.50	5.518
7	7.38	2.44	1.037
8	7.38	2.53	10.253
9	7.32	2.46	1.027
10	7.31	2.53	35.328
11	7.35	2.44	1.184
12	7.36	2.57	58.145
13	7.36	2.46	0.969
14	7.33	2.57	69.985
15	7.35	2.45	3.926
16	7.38	2.46	4.133
17	7.31	2.46	9.266

**Table 2:** Chemical reagents and raw materials for laboratory use.

Chemical Formula of Reagent/Raw Materials	Specifications	Manufacturer	City
Dodecamine	Analytical grade	McLean Reagents Ltd.	Shanghai, China
Ethylenediamine	Analytical grade	McLean Reagents Ltd.	Shanghai, China
chloroethylsulphonic acid	Analytical grade	McLean Reagents Ltd.	Shanghai, China
γ-(2,3-epoxypropyl) trimethylsilane	Analytical grade	Dongguan Kangjin New Materials Technology Co., Ltd.	Dongguan, China
Nano-SiO_2_	15~30 nm	Shanghai Aladdin Biochemical Technology Co., Ltd.	Shanghai, China

**Table 3:** Foam properties and surface tension in different formulation systems.

Formulation Name	Volume of Foam (mL)	Half-Life (min)	Foam Complexity Index (mL·min)	Surface Tension (×10^−2^ mN/m)
0.4% LS-12	180	70	9450	10.13
0.1% LT-14	150	65	7312.5	9.38
0.4% LS-12 + 0.1% LT-14	200	80	12,000	6.63
0.4% LS-12 + 0.1% LT-14 + 0.03%WB	200	100	15,000	6.46

**Table 4:** Comparison of static imbibition effects in different core materials.

Core No.	Permeability/mD	Fluid	Water Contact Angle/°	Total EOR /%
15	3.926	formation water	39.6	24.50
16	4.133	LIFT-NSF	17.0	28.88
17	9.266	LIFT-NSF	17.0	67.63

**Table 5:** Results of sealing performance evaluation.

Core No.	Air Permeability (mD)	Initial Aqueous Phase Permeability (mD)	Subsequent Water-Driven Permeability (mD)	Resistance Coefficient	Residual Resistance Coefficient
4	0.505	0.341	0.053	1.086	2.241
5	1.003	0.626	0.140	6.582	4.457
6	5.158	3.185	0.794	15.468	4.013

**Table 6:** Effect of injection pressure differential or displacement rate on oil recovery.

Core No.	Injection Pressure/MPa	Injection Rate/mL·min^−1^	Water Displacement Recovery Rate/%	Foam Displacement Recovery Rate/%	Subsequent Water-Driven Recovery Rate/%	Total EOR/%
1	4	0.0213~0.1866	39.41	6.04	14.30	59.75
2	5	0.0135~0.0518	39.97	8.18	13.19	61.34
3	6	0.0087~0.0276	40.35	1.87	18.22	60.44

**Table 7:** Effect of foam plug volume on oil recovery.

Core No.	Injection Pressure/MPa	Foam Plug Volume/PV	Water Displacement Recovery Rate/%	Foam Displacement Recovery Rate/%	Subsequent Water-Driven Recovery Rate/%	Total EOR/%
1	5	0.15	38.53	1.73	19.06	59.32
2		0.20	39.97	8.18	13.19	61.34
3		0.30	39.31	9.85	19.53	68.69
5		0.60	39.66	4.52	12.77	56.95

**Table 8:** Results of oil displacement experiments in heterogeneous media.

Core No.	Permeability/mD	Grade Difference	Water-Flood Recovery Rate/%			Foam and Subsequent Water-Flooding Recovery Rate/%			Total EOR /%		
			Hypotonic	Hyperosmotic	General	Hypotonic	Hyperosmotic	General	Hypotonic	Hyperosmotic	General
7	1.037	10	0	60.72	35.03	6.54	13.38	10.48	6.54	74.10	45.51
8	10.235										
9	1.027	35	0	63.61	26.21	2.14	11.69	6.07	2.14	75.30	32.28
10	35.328										
11	1.184	50	0	72.46	46.55	1.76	9.33	6.62	1.76	81.79	53.17
12	58.145										
13	0.969	60	0	56.19	37.89	0	28.53	19.24	0	84.72	57.13
14	65.985										

## Data Availability

The original contributions presented in this study are included in the article. Further inquiries can be directed to the corresponding author(s).
